# Atherogenic index of plasma is a novel and strong predictor associated with fatty liver: a cross-sectional study in the Chinese Han population

**DOI:** 10.1186/s12944-019-1112-6

**Published:** 2019-09-12

**Authors:** Fangfei Xie, Hong Zhou, Yun Wang

**Affiliations:** 1 Physical Examination Center, the Affiliated Suzhou Hospital of Nanjing Medical University, Suzhou, Jiangsu People’s Republic of China; 2 Suzhou Health Management Society, Suzhou, Jiangsu People’s Republic of China

**Keywords:** Fatty liver, Atherogenic index of plasma, Predictor

## Abstract

**Background:**

Atherogenic index of plasma (AIP) has been reported to be an important predictor for coronary artery disease and obesity. However, few studies has yet systematically evaluated the association between AIP and Fatty Liver (FL) and its advantage in FL prediction compared with BMI, waist, SBP, DBP, BG, ALT and AST.

**Methods:**

A total of 7838 participants aged from 19 to 93 years were included in this study. Height, weight, waist, SBP, DBP, BG, ALT and AST were measured. Difference analyses, odds ratio calculation, logistic and predictive analyses were used to evaluate the association and discrimination ability between AIP, BMI, waist, SBP, DBP, BG, ALT, AST and FL.

**Results:**

Compared with non-FL, AIP in FL people showed a significant increase. Subjects in the higher quartiles of AIP had a significantly increased risk of fatty liver compared with those in the lowest quartile (*P* < 0.01) after adjustment of gender and age. ORs were grown faster in female and youth group. AIP contributed most in the logistic eq. (B = 2.64, *P* < 0.01) and showed high ability in risk prediction for FL (AUC = 0.810, P < 0.01).

**Conclusions:**

AIP might be a novel and strong predictor associated with FL in Chinese Han population. Higher AIP level was positively and strongly associated with FL.

**Electronic supplementary material:**

The online version of this article (10.1186/s12944-019-1112-6) contains supplementary material, which is available to authorized users.

## Background

Fatty liver (FL) has become a global problem of public health over the past few decades with prevalence nearly 25% and medical burden in both developed and developing countries [1, 2]. The prevalence of Nonalcoholic fatty liver disease (NAFLD) is 15–30% in China based on studies conducted over the past 3 years [3]. It is a common cause of chronic liver disease associated with an increased risk of cirrhosis and hepatocellular carcinoma, through its progression to steatohepatitis and fibrosis [4–6]. Chronic liver disease prevalence in adolescent and young adult has more than doubled over the past three decades mainly due to rise in NAFLD prevalence [7]. In the last decades, an alarming increase in the prevalence of FL has been observed, along with increasing rates of obesity [8, 9]. Research found the increasing obesity epidemic has contributed to the rising prevalence of NASH-associated endstage liver disease in the US [10]. Although genetic factors contribute toward the development of NASH, obesity and MS remain the leading causes [11].

Looking for a blood lipid index to be used as a biomarker for FL will make up for the limitation of regular indexes, such as, Glutamic-pyruvic transaminase (ALT) and Glutamic-oxalacetic transaminase (AST). Besides, the lipid biomarker as a direct biological target will also be helpful in FL prevention and therapeutic treatment. Atherogenic index of plasma (AIP) is a novel index composed of triglycerides and high-density lipoprotein cholesterol which consider two lipid compounds comprehensively [12]. It has been used to quantify blood lipid levels and commonly used as optimal indicator of dyslipidemia, obesity, and cardiovascular diseases [13–15]. Qian Wang et al. found that AIP were strongly correlated with NAFLD in obese participants and suggested to be used as a regular monitoring index of NAFLD for obese men [16]. However, whether AIP is related to the risk of FL in the Chinese Han population including obese men remains unknown. Therefore, we explored the relationship between AIP and FL in a large-scale Chinese population and evaluated the predictive ability of AIP for FL compared with other compounds.

## Methods

### Study population

The study subjects were recruited in physical examination Center of Suzhou in southeast of China, during January 2018 to December 2018. Participants in this study were Chinese Han ethnicity ageing from 18 to 93 years. After excluding subjects for lacking data, a total of 7838 subjects were finally included in the analysis. The study was approved by the Affiliated Suzhou Hospital of Nanjing Medical University and all subjects agreed to participate into the present study.

### Data collection

Health examination was performed in the morning after the examinees fasted overnight. Anthropometric indices were measured by an eligible physician. Weight and height were measured in light indoor clothing without shoes and heavy clothes, using a calibrated measuring apparatus. Body Mass Index (BMI) was calculated as body weight (in kilograms) divided by the square of height (in meters). Waist refers to the horizontal circumference through the umbilical point of the measured standing person. Blood pressure was measured with automatic blood pressure meter after 10-min rest in the sitting room. Peripheral blood was drawn into an EDTA-containing tube and was subjected to biochemical experiments within 3 h. Hexokinase method was used to detect FBG. Blood lipid indexes including Triglyceride (TG) and High density lipoprotein (HDL) and liver function index including ALT and AST were measured by Beckman AU5800 autoanalyzer. AIP was calculated as logarithmic transformation of the ratio of TG to HDL. FL was defined with liver ultrasonography showing steatosis by an eligible sonologists.

### Statistical analysis

Firstly, all participants were divided into FL and non-FL groups. The baseline variables (gender, age, BMI, waist, SBP, DBP, BG, ALT, AST and AIP) were compared using the Chisqure tests and Rank tests appropriately. Total participants were further categorized into four groups according to the quartiles of AIP (≤ − 0.2109, − 0.2105 to − 0.0147, − 0.0144 to 0.2081, ≥0.2083). The odds ratio (OR) of FL were estimated for higher three categories of AIP with the lowest one as a reference. Univariate logistic regressions and multivariate logistic regression analyses were conducted to evaluate the association between AIP, FL and other indicators. Area under the curve (AUC) of receiver operating characteristic (ROC) was calculated to compare the predictive value between AIP and other indicators for predicting FL. All statistical analyses were performed with the Statistical Package for the Sciences (SPSS, version 17.0). A value of *P* < 0.01 in two-tailed test was considered significant.

## Results

A total of 7838 subjects were included in our study, including 1919 (24.48%) FL patients and 5919 (75.52%) controls. The mean age of patients was 45.8 ± 13.0 (age range: 19–90), 75.77% (1454) of them were male. The mean age of controls was 43.4 ± 13.6 (age range: 19–93), 44.16% (2614) of them were male. As shown in Table 1, participants with FL were more likely to have higher BMI, waist, SBP, DBP, BG, ALT, AST and AIP. In Table 2, the participants with higher AIP tended to be male, younger and have higher BMI, waist, SBP, DBP, BG, ALT and AST.

**Table 1:** IQR and Difference between FL and Non-FL Group among Total Population

	Fatty liver			Non-fatty liver			*P* value
	25%	50%	75%	25%	50%	75%	
Age, years	36	44	55	32	41	53	< 0.01
Body Mass Index (BMI), kg/(m^2^)	24.80	26.67	28.73	20.83	22.84	24.97	< 0.01
Waist, cm	87	92	97	74	80	87	< 0.01
Systolic blood pressure (SBP), mmHg	121	132	145	111	121	134	< 0.01
Diastolic blood pressure (DBP), mmHg	73	80	88	66	73	81	< 0.01
Blood glucose (BG), mmol/l	5.07	5.41	5.96	5.07	5.41	5.96	< 0.01
Glutamic-pyruvic transaminase (ALT), U/L	19	28	43	19	28	43	< 0.01
Glutamic-oxalacetic transaminase (AST), U/L	19	24	30	19	24	30	< 0.01
Atherogenic index of plasma (AIP)	0.07	0.25	0.42	−0.26	−0.09	0.10	< 0.01

**Table 2:** Baseline Characteristics in Four Groups According to AIP Quartile among Total Population

	Quartile 1 (−∞, − 0.2109]	Quartile 2 [− 0.2105, − 0.0147]	Quartile 3 [− 0.0144, 0.2081]	Quartile 4 [0.2083, +∞]	*P* value
Number	1961	1958	1960	1959	
Male, n(%)	493 (25.14%)	896 (45.76%)	1176 (60%)	1503 (76.72%)	< 0.01
Age, years	40.33 ± 12.88	43.28 ± 13.86	46.09 ± 13.74	46.32 ± 12.68	< 0.01
Body Mass Index (BMI), kg/(m2)	21.68 ± 2.68	23.36 ± 3.12	24.80 ± 3.16	26.25 ± 3.29	< 0.01
Waist, cm	75.86 ± 7.78	81.41 ± 8.92	86.09 ± 8.69	90.41 ± 8.43	< 0.01
Systolic blood pressure (SBP), mmHg	119.27 ± 17.27	124.84 ± 18.47	128.53 ± 132.84	132.84 ± 19.20	< 0.01
Diastolic blood pressure (DBP), mmHg	71.59 ± 10.75	74.45 ± 11.32	76.96 ± 11.81	80.57 ± 11.63	< 0.01
Blood glucose (BG), mmol/l	5.16 ± 0.76	5.32 ± 0.88	5.48 ± 1.14	5.75 ± 1.50	< 0.01
Glutamic-pyruvic transaminase (ALT), U/L	15.92 ± 13.78	19.84 ± 16.01	23.67 ± 19.25	32.57 ± 26.22	< 0.01
Glutamic-oxalacetic transaminase (AST), U/L	20.04 ± 7.84	21.51 ± 10.19	22.57 ± 10.25	25.96 ± 13.22	< 0.01

Note: Variables are expressed as the mean ± standard deviation

In Table 3, participants in higher AIP quartiles all had a significantly increased risk of FL compared with the lowest group with the ORs of 5.38, 14.0 and 46.9, respectively (*P* < 0.01). After adjustment for gender and age, the associations remained significant (*P* < 0.01) (Table 4). Independent of age, subjects in the higher quartiles of AIP have higher risk of FL.

**Table 3:** ORs for FL in Four Groups According to AIP Quartile among Total Population

	AIP				
	Quartile 1 (−∞, −0.2109]	Quartile 2 [−0.2105, −0.0147]	Quartile 3 [− 0.0144, 0.2081]	Quartile 4 [0.2083, +∞]	Total
Fatty liver	51	246	533	1089	1919
Non-fatty liver	1910	1712	1427	870	5919
Total	1961	1958	1960	1959	7838
OR		5.38(P < 0.01)	14.0(*P* < 0.01)	46.9(*P* < 0.01)	

Note: OR was calculated for the risk of FL in Quartile 2, Quartile 3 and Quartile 4 compared with the Quartile 1 separately

**Table 4:** ORs for FL in Four Groups According to AIP Quartile after adjustment of gender and age

ORs for FL in Four Groups According to AIP Quartile after adjustment of gender										
	Male					female				
	Quartile 1	Quartile 2	Quartile 3	Quartile 4	Total	Quartile 1	Quartile 2	Quartile 3	Quartile 4	Total
Fatty liver	29	152	378	895	1454	22	94	155	194	465
Non-fatty liver	464	744	798	608	2614	1446	968	629	262	3305
Total	493	896	1176	1503	4068	1468	1062	784	456	3770
OR		3.27 (*P* < 0.01)	7.58 (*P* < 0.01)	23.6 (*P* < 0.01)			6.38 (*P* < 0.01)	16.2 (*P* < 0.01)	48.7 (*P* < 0.01)	
ORs for FL in Four Groups According to AIP Quartile after adjustment of age										
	< 45 years					> = 45 years				
	Quartile 1	Quartile 2	Quartile 3	Quartile 4	Total	Quartile 1	Quartile 2	Quartile 3	Quartile 4	Total
Fatty liver	28	124	240	581	973	28	128	291	512	959
Non-fatty liver	1288	1029	716	369	3402	615	679	711	499	2500
Total	1316	1153	956	950	4375	643	807	1002	1011	3463
OR		5.54 (*P* < 0.01)	15.4 (*P* < 0.01)	72.4 (*P* < 0.01)			4.14 (*P* < 0.01)	8.99 (*P* < 0.01)	22.5 (*P* < 0.01)	

Note: OR was calculated for the risk of FL in Quartile 2, Quartile 3 and Quartile 4 compared with the Quartile 1 separately

**Fig. 1:**
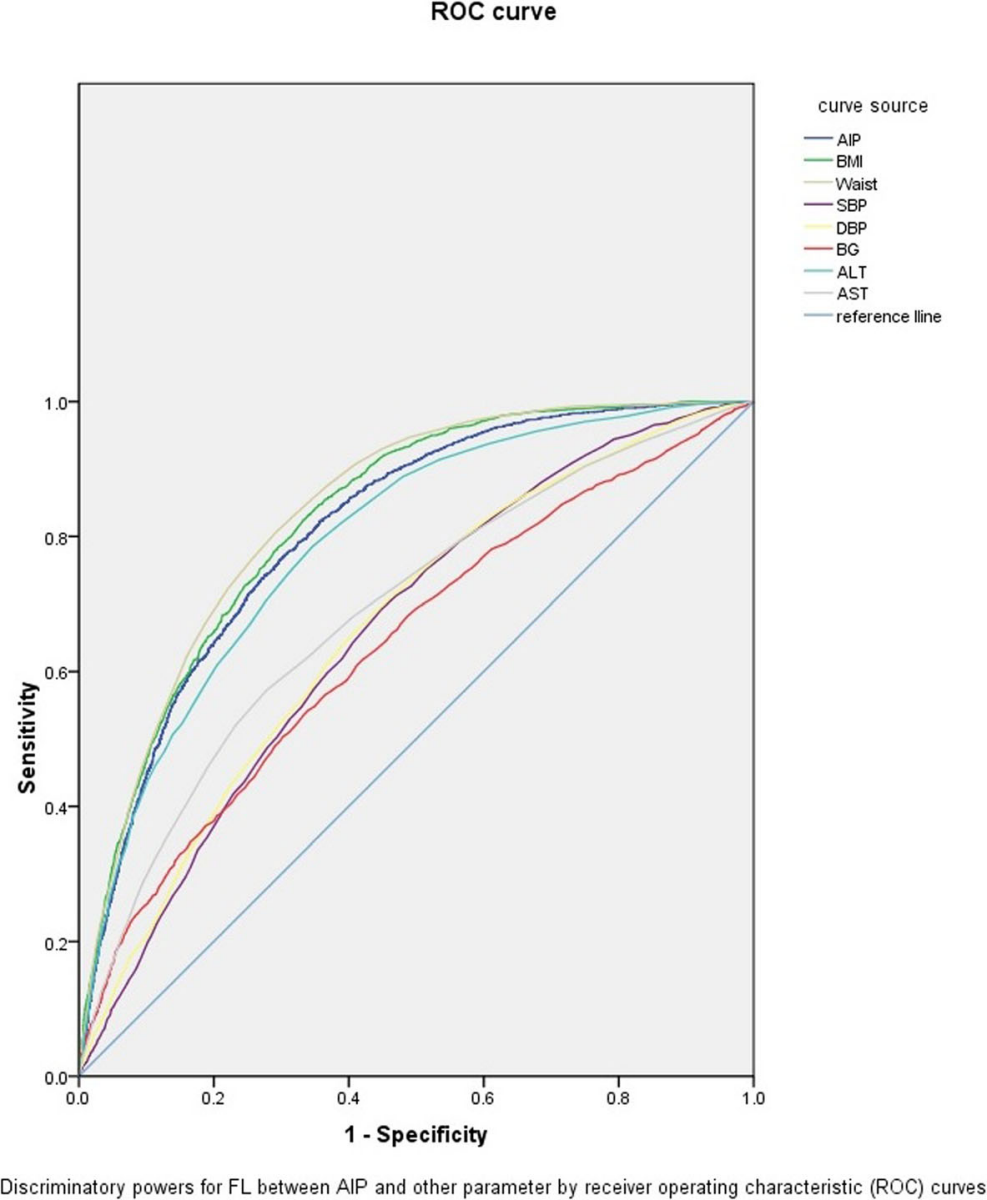
ROC curves of eight variables

As shown in Additional file 1: Table S1 univariate logistic regressions shown significant association (*P* < 0.05) between FL and BMI, waist, SBP, DBP, BG, ALT, AST, AIP separately. In multivariate logistic regression analysis, AIP was the parameter represented for lipid components which contributed most to FL with B of 2.638 (P < 0.01) compared with other biomarkers in Table 5. In addition, AIP had a higher risk of FL with the OR of 13.992 compared with other parameters. In Table 6 and Fig. 1, AIP showed high risk predictive ability for FL (AUC = 0.810, 95%CI:0.800~0.820, *P* < 0.01).

**Table 5:** Logistic Regression Analysis for FL among Total Population

Variables	Beta	Stand error	OR	*P*
Body Mass Index (BMI), kg/(m2)	0.160	0.019	1.173	< 0.01
Waist, cm	0.058	0.007	1.059	< 0.01
Systolic blood pressure (SBP), mmHg	0.002	0.003	1.002	0.479
Diastolic blood pressure (DBP), mmHg	0.005	0.004	1.005	0.186
Blood glucose (BG), mmol/l	0.160	0.028	1.174	< 0.01
Glutamic-pyruvic transaminase (ALT), U/L	0.032	0.003	1.033	< 0.01
Glutamic-oxalacetic transaminase (AST), U/L	−0.022	0.006	0.978	< 0.01
Atherogenic index of plasma (AIP)	2.638	0.135	13.992	< 0.01
Constant	−12.232	0.445		< 0.01

**Table 6:** AUC in ROC curve among Total Population

Variables	AUC	Stand error	95% CI
Body Mass Index (BMI), kg/(m2)	0.826	0.005	(0.817, 0.836)
Waist, cm	0.835	0.005	(0.826, 0.845)
Systolic blood pressure (SBP), mmHg	0.660	0.007	(0.646, 0.673)
Diastolic blood pressure (DBP), mmHg	0.665	0.007	(0.646, 0.673)
Blood glucose (BG), mmol/l	0.639	0.007	(0.625, 0.654)
Glutamic-pyruvic transaminase (ALT), U/L	0.792	0.006	(0.781, 0.803)
Glutamic-oxalacetic transaminase (AST), U/L	0.689	0.007	(0.676, 0.703)
Atherogenic index of plasma (AIP)	0.810	0.005	(0.800, 0.820)

## Discussion

In this large-sample cross-sectional survey, we explored the relationship between AIP and FL risk and found that subjects with higher AIP levels tended to have a higher risk of FL. AIP showed a strongest relationship (B = 2.638, OR = 13.992, P < 0.01) with FL compared other parameters in multivariate logistic regression analysis. Besides, ORs were grown faster in female and youth group.

The association of obesity with FL has been established in a few studies [6, 8, 17, 18]. Epidemiological studies propose a causative link between obesity and progressive liver disease in individuals [17, 18]. Obesity has been linked not only to initial stages of the disease, but also to its severity [8]. The pathophysiology and clinical study had also shown the progression of FL results from an imbalance between lipid uptake and lipid disposal and eventually causes oxidative stress and hepatocyte injury [10]. Dobiasova M and Frohlich J et al. had proposed AIP has a stronger sensitivity that reflects the interaction between atherogenic and protective lipoprotein [12]. Compared with the traditional lipid profile, the integration of the two indies (TG and HDL) to generate a composite one of AIP for FL could avoid the inconsistent assessment of different lipid components. Although a previous study reported a higher prevalence of NAFLD associated with AIP elevating, it is limited to obese people and did not taken blood pressure into account which is independently associated with NAFLD [16, 19].

Our study found that there were concordances between increased AIP and significant increase in the value of BMI, waist, BG, ALT, AST, which agreed with the results described by Qian Wang et al. Besides, SDP and DBP also showed an increase with AIP, whereas increased blood pressure is related to the development of fatty liver disease and the possible subsequent progression to liver fibrosis. Insulin resistance and activation of the renin-angiotensin-aldosterone system (RAAS) might provide potential pathophysiologic links between these clinical entities [20].

We calculated ORs removed influence carried by gender and age and found an increasing risk with rising AIP [21, 22]. Besides, ORs in female and people under 45 years old group shown a faster growth rate compared with male and people upon 45 years old group. Although FL is primarily a male disease, the alteration in sex hormone levels, specifically reduced estrogens and increased androgens during and after menopause, is an important factor in the emergence of FL [23]. Cai M-J et al. found the proportion of male patients gradually decreased with age, while the proportion of female patients increased [24]. Compared with male and old, female and youth have excessive intake of calories combined with poor physical activity which may be responsible for the accumulation of intrahepatic fat and hepatotoxicity [23, 25].

In our study, the Multivariate logistic analyses explored the contribution and found AIP (B = 2.638, OR = 13.992, *P* < 0.01) was the strongest biomarker for FL compared with other parameters. Although correlation exist in hypertension, diabetes, hepatopathy and FL, it is been widely accepted that lipid accumulation in the liver is the prerequisite for NAFLD [5, 8, 20, 26]. With AIP, a comprehensive index, lipid could be adjusted for the lack of the inconsistent assessment of different lipid components and simplify the prediction task in practical application. Previous studies have shown AIP was a better predictor of NAFLD than LDL and TC, so we conducted a Multivariate analysis with AIP in place of TG, TC, LDL, HDL [16]. Besides BMI and waist, AIP showed a better AUC in predicting FL than any other indexes. The AUC of BMI (0.826) and waist (0.835) was larger than that of AIP (0.810), which may be explained by the fact that some obese people have FL even in normal blood lipid.

Our study has several advantages that deserved mentioning. Firstly, it is a large-scale survey in China for the relationship between FL and AIP. Secondly, AIP is generated by two substances (TG and HDL-C) in blood, avoiding the inconsistent assessment of different lipid components and simplify the prediction task in practical application.

There are also limitations should be mentioned. At first, we could not obtain data of confounders including diet, physical activity as well as alcohol history. These data are associated with FL and influenced the accuracy of results. Secondly, although biological association between AIP and FL exists, cross-sectional study have difficult in building causal relationship.

## Conclusion

In summary, AIP is a novel and strong predictor associated with FL. Higher AIP was positively with FL. It can be used as a reference index in diagnosis and treatment of FL.

## Additional file

Additional file 1: **Table S1.** univariate logistic regressions for FL and BMI, waist, SBP, DBP, BG, ALT, AST, AIP among Total Population. (DOCX 17 kb)

## Data Availability

The datasets during and/or analyzed during the current study are available from the corresponding author on reasonable request.

## Abbreviations

AIP: Atherogenic Index of Plasma
ALT: Glutamic-pyruvic transaminase
AST: Glutamic-oxalacetic transaminase
AUC: Area Under the Curve
BG: Blood Glucose
BMI: Body Mass Index
DBP: Diastolic Blood Pressure
FL: Fatty Liver
HDL: High Density lipoprotein
OR: Odds Ratio
ROC: Receiver Operating Characteristic
SBP: Systolic Blood Pressure
TG: Triglyceride
