# Supplementary material for: Atherogenic index of plasma is a novel and strong predictor associated with fatty liver: a cross-sectional study in the Chinese Han population
Source: Lipids Health Dis. 2019 Sep 12;18:170. doi: 10.1186/s12944-019-1112-6 (PMC6739922; doi:10.1186/s12944-019-1112-6)
Supplement: Supplementary file 1 — Table S1. univariate logistic regressions for FL and BMI, waist, SBP, DBP, BG, ALT, AST, AIP among Total Population. (DOCX 17 kb) [file 12944_2019_1112_MOESM1_ESM.docx]

**Additional file 1: Table 1: univariate logistic regressions for FL and BMI, waist, SBP, DBP, BG, ALT, AST, AIP among Total Population**

| **Variables** | **Beta** | **P** |
| --- | --- | --- |
| **Body Mass Index (BMI), kg/(m2)** | 0.40 | <0.01 |
| **Waist, cm** | 0.15 | <0.01 |
| **Systolic blood pressure (SBP), mmHg** | 0.03 | <0.01 |
| **Diastolic blood pressure (DBP), mmHg** | 0.05 | <0.01 |
| **Blood glucose (BG), mmol/l** | 0.40 | <0.01 |
| **Glutamic-pyruvic transaminase (ALT), U/L** | 0.05 | <0.01 |
| **Glutamic-oxalacetic transaminase (AST), U/L** | 0.06 | <0.01 |
| **Atherogenic index of plasma (AIP)** | 4.24 | <0.01 |
